# Noncardiogenic Pulmonary Edema After Neostigmine Administration During Emergence From General Anesthesia: A Case Report

**DOI:** 10.1155/cria/8828001

**Published:** 2026-05-27

**Authors:** Jean-Claude Stephan, Mikhael Kossaify, Souheil Chamandi, Anthony Kanbar

**Affiliations:** ^1^ Department of Anesthesiology and Critical Care, Notre Dame des Secours University Hospital Center, Street 93 Postal Code 3, Byblos, Lebanon; ^2^ School of Medicine and Medical Sciences, Holy Spirit University of Kaslik, P.O. Box 446, Jounieh, Lebanon, usek.edu.lb

**Keywords:** acetylcholinesterase inhibitor, case report, drug adverse effect, neostigmine, noncardiogenic pulmonary edema

## Abstract

We report the case of a previously healthy 27‐year‐old man who developed acute pulmonary edema shortly after receiving neostigmine‐atropine for neuromuscular blockade reversal during appendectomy under general anesthesia. Before extubation, he developed severe hypoxemia, increased airway pressures, pink frothy tracheal secretions, and bilateral diffuse pulmonary infiltrates on chest radiography. Cardiogenic pulmonary edema, fluid overload, negative‐pressure pulmonary edema, and perioperative hypersensitivity were considered unlikely based on the clinical findings, bedside cardiac ultrasound, limited intraoperative fluids, normal histamine and tryptase levels, and negative skin testing. The event was therefore considered a diagnosis of exclusion temporally associated with neostigmine administration. This case highlights the importance of careful differential diagnosis and cautious causal attribution when respiratory compromise occurs after reversal.

## 1. Background

Neuromuscular blocking drugs (NMBD) induce muscle relaxation by blocking acetylcholine receptors at the neuromuscular junction. Neostigmine is commonly used for the reversal of nondepolarizing neuromuscular blockade [1]. Although widely used and generally considered safe, anaphylaxis and cardiovascular collapse have been reported [2].

We report a rare case of acute postoperative noncardiogenic pulmonary edema (NCPE) temporally associated with neostigmine administration in a patient who underwent appendectomy. Written informed consent for publication of this case and accompanying clinical details was obtained from the patient. According to institutional policy, ethics committee approval was waived for publication of a single case report. This case report was prepared in accordance with the CARE reporting guideline, and a completed CARE checklist is provided as supporting information (available here).

## 2. Case Report

A 27‐year‐old previously healthy man weighing 81 kg was scheduled for an appendectomy. He smoked occasionally and did not have any previous surgical history or allergies to food, medications, or latex. The preanesthesia examination did not reveal any specific findings. No premedication was administered. On arrival to the operating room, standard monitoring revealed a blood pressure (BP) of 123/68 mmHg, a heart rate (HR) of 78 beats/min^−1^, and a peripheral oxygen saturation (SpO_2_) of 97% on room air. After adequate preoxygenation, anesthesia was induced with propofol 1% (2 mg·kg^−1^) and fentanyl (2 mcg/kg^−1^). After achieving a complete loss of consciousness, rocuronium (1 mg·kg^−1^) was given, and tracheal intubation was performed and confirmed by bilateral breath sounds and capnography. Sevoflurane 1 vol% was administered for the maintenance of anesthesia. During the surgery, the patient was hemodynamically stable. He received 450 mL of Ringer solution. SpO_2_ was maintained at 99%. Airway pressure (Paw) varied between 11 and 13 mmHg with a tidal volume (Vt) of 450 mL. The procedure lasted approximately 50 min and was concluded without complications.

**FIGURE 1:**
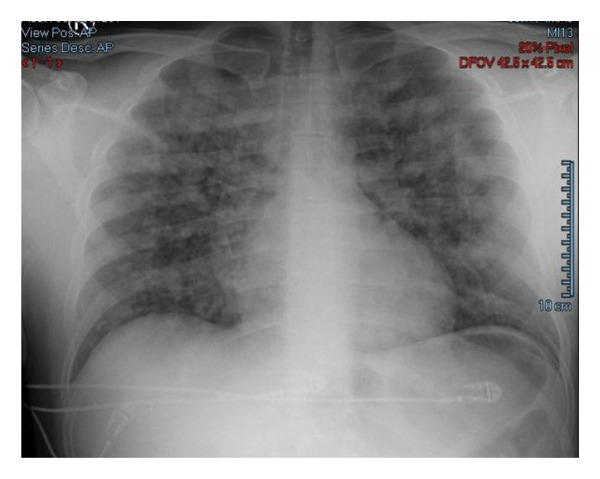
Chest radiograph showing diffuse bilateral pulmonary infiltrates compatible with acute pulmonary edema. Arrows show increased pulmonary vascular congestion.

Neuromuscular recovery was assessed using quantitative train‐of‐four monitoring at the ulnar nerve/adductor pollicis. A TOF ratio of 0.9 was documented at the end of surgery, after which neostigmine (0.04 mg kg^−1^) and atropine (0.02 mg kg^−1^) were administered for reversal. Six minutes later, and before extubation, the patient suddenly developed hypertension, tachycardia, and hypoxemia (PaO_2_/FiO_2_ ratio < 100 mmHg). The ventilator monitor showed extremely high airway pressure and significantly decreased tidal volume, which limited its function. The patient was sedated and transitioned to controlled ventilation. Chest auscultation revealed bilateral crackles. Copious pink frothy secretions were suctioned through his tracheal tube. Diffuse bilateral patchy infiltrates, homogenously distributed throughout the lungs, were observed on chest X‐ray (Figure 1). The electrocardiogram showed sinus tachycardia and the cardiac ultrasound, which was performed in the operating room, did not reveal any structural cardiac pathology. Blood samples taken 30 min postdesaturation showed normal histamine (0.4 ng·mL^−1)^ and tryptase (0.9 μg·L^−1^) levels.

Based on the clinical and radiographic findings, acute postoperative pulmonary edema was diagnosed. The sequence of key perioperative events is summarized in Table 1. He was shifted to the intensive care unit, and the respiratory failure was treated using diuretics (Furosemide 80 mg I.V.). Gradual weaning of the mechanical ventilation was achieved, and the patient was extubated 12 h later without any sequelae. Skin prick tests performed with neostigmine were negative.

**TABLE 1:** Timeline of clinical events.

Stage/timepoint	Clinical event	Key findings/actions
Preoperative	27‐year‐old previously healthy man scheduled for appendectomy	Occasional smoker; no prior surgery; no known allergy to food, medications, or latex
Operating room arrival	Baseline assessment	BP 123/68 mmHg, HR 78 beats/min, SpO_2_ 97% on room air
Induction of anesthesia	General anesthesia induced	Propofol 2 mg/kg, fentanyl 2 μg/kg, rocuronium 1 mg/kg
Airway management	Tracheal intubation	Confirmed by bilateral breath sounds and capnography
Intraoperative period	Stable surgery course	Hemodynamically stable; SpO_2_ 99%; airway pressure 11–13 cm H_2_O; tidal volume 450 mL; 450 mL Ringer solution administered
End of surgery	Neuromuscular recovery assessed	Quantitative train‐of‐four monitoring at ulnar nerve/adductor pollicis showed TOF ratio 0.9
Reversal	Reversal agents administered	Neostigmine 0.04 mg/kg and atropine 0.02 mg/kg
6 min after reversal, before extubation	Acute respiratory deterioration	Hypertension, tachycardia, severe hypoxemia (PaO_2_/FiO_2_ < 100), markedly increased airway pressure, reduced tidal volume
Immediate respiratory findings	Pulmonary edema suspected	Bilateral crackles, copious pink frothy tracheal secretions, diffuse bilateral infiltrates on chest X‐ray
Immediate evaluation	Differential diagnosis work‐up	ECG: sinus tachycardia; bedside cardiac ultrasound in OR: no structural cardiac pathology; histamine 0.4 ng/mL; tryptase 0.9 μg/L
ICU management	Supportive treatment	Controlled ventilation, ICU transfer, furosemide 80 mg IV
Recovery	Clinical improvement	Gradual ventilator weaning; extubated 12 h later without sequelae
Follow‐up allergy assessment	Hypersensitivity evaluation	Skin prick testing with neostigmine negative

## 3. Discussion

Pulmonary edema in the perioperative period is an uncommon but potentially serious cause of acute respiratory failure [3, 4]. In the postoperative setting, the main differential diagnoses include cardiogenic pulmonary edema, fluid overload, negative‐pressure pulmonary edema, perioperative hypersensitivity, and drug‐associated NCPE [4].

In the present case, acute respiratory deterioration occurred within minutes of neostigmine‐atropine administration and before extubation. In everyday anesthesia practice, reversal agents are commonly administered routinely at the end of surgery. Therefore, respiratory deterioration occurring within minutes of reversal, particularly before extubation while the airway remains secured, is clinically significant and should prompt consideration of reversal‐associated complications in the differential diagnosis. The clinical presentation was characterized by severe hypoxemia, increased airway pressure, reduced tidal volume, bilateral crackles, copious pink frothy tracheal secretions, and diffuse bilateral pulmonary infiltrates on chest radiography. However, neostigmine‐associated NCPE should be regarded as a diagnosis of exclusion and a temporal association rather than definitive proof of direct causality [4–8].

Cardiogenic pulmonary edema was considered unlikely because the patient remained hemodynamically stable throughout the procedure, electrocardiography showed only sinus tachycardia, and bedside echocardiographic evaluation performed in the operating room by a cardiologist did not reveal structural or functional findings suggestive of acute cardiac failure. Cardiac biomarkers were not obtained. Therefore, subtle myocardial dysfunction cannot be completely excluded, although no intraoperative electrocardiographic or bedside echocardiographic findings suggested acute cardiac failure. Fluid overload was also unlikely given the limited intraoperative fluid administration of 450 mL of Ringer solution during a short and uncomplicated procedure [9].

Negative‐pressure pulmonary edema was considered less likely because the respiratory event occurred before extubation while the airway remained secured with an endotracheal tube, and no documented episode of laryngospasm or upper airway obstruction was observed [3, 10]. Perioperative hypersensitivity was another important consideration [2]. However, normal serum histamine and tryptase levels, together with negative skin prick testing to neostigmine, made an immediate hypersensitivity mechanism less likely. This more detailed allergy work‐up also strengthens the argument against an immediate hypersensitivity mechanism compared with several previous reports. Atropine was considered less likely to be the primary contributor. Atropine is a competitive muscarinic antagonist that is routinely coadministered with neostigmine to offset muscarinic adverse effects such as bradycardia and increased secretions. Similar cases of postoperative NCPE have also been reported after reversal with neostigmine‐glycopyrrolate rather than atropine, and there is no strong evidence that atropine itself is an independent cause of NCPE in this setting [2, 6].

Given the close temporal relationship between reversal administration and respiratory deterioration, drug‐associated NCPE temporally related to neostigmine administration was considered the most plausible working diagnosis after exclusion of the more common alternative etiologies. Similar cases have been reported previously; therefore, the present case is not intended as the first description of this phenomenon. In addition to previously published case reports [5–8], Sasaki et al. reported that high‐dose or unwarranted neostigmine use was associated with increased postoperative respiratory morbidity, including atelectasis, pulmonary edema, and reintubation. In the present case, the administered dose was not excessive, and quantitative neuromuscular monitoring was available; rather, the clinically relevant feature was administration after a documented train‐of‐four ratio of 0.9. The main contribution of this case lies in the structured differential diagnosis and the onset of respiratory deterioration before extubation while the airway remained secured.

An important feature of this case is that neostigmine was administered after a documented quantitative train‐of‐four ratio of 0.9. Because a TOF ratio of 0.9 is generally considered consistent with adequate neuromuscular recovery, pharmacologic antagonism may not have been necessary at that stage. This timing is therefore clinically relevant. Experimental work suggests that administration of acetylcholinesterase inhibitors after near‐complete recovery may impair upper airway muscle function and contribute to postoperative respiratory compromise [11]. Although this mechanism does not fully explain the present case, it provides a possible physiologic link between neostigmine administration at near‐complete recovery and the subsequent respiratory event. Nevertheless, causality cannot be established from a single case report, and the exact mechanism remains speculative [11, 12].

In conclusion, we describe a rare but previously reported episode of acute postoperative NCPE temporally associated with neostigmine administration. The diagnosis relied on exclusion of more common alternative causes, including cardiogenic pulmonary edema, fluid overload, negative‐pressure pulmonary edema, and perioperative hypersensitivity. This case highlights the importance of structured differential diagnosis, careful interpretation of neuromuscular monitoring, and cautious attribution of causality when respiratory deterioration occurs after reversal of neuromuscular blockade [13].

**Nomenclature**
NMBD: Neuromuscular blocking drugs
NCPE: Noncardiogenic pulmonary edema
BP: Blood pressure
HR: Heart rate
SpO_2_
: Peripheral oxygen saturation
Paw: Airway pressure
PE: Pulmonary edema

## Author Contributions

Jean‐Claude Stephan: writing the background, case report, and discussion and literature review.

Souheil Chamandi and Mikhael Kossaify: discussion of the manuscript and review.

Anthony Kanbar: participation in the discussion writing and literature review.

## Funding

No financial funding for this article.

## Conflicts of Interest

The authors declare no conflicts of interest.

## Supporting Information

Additional supporting information can be found online in the Supporting Information section.

## Supporting information


**Supporting Information** Supporting File 1: CARE checklist. Completed CARE checklist for the case report entitled “Noncardiogenic Pulmonary Edema After Neostigmine Administration During Emergence From General Anesthesia: A Case Report.”

## Data Availability

The data that support the findings of this study are available from the corresponding author upon reasonable request.
