# Supplementary material for: Noncardiogenic Pulmonary Edema After Neostigmine Administration During Emergence From General Anesthesia: A Case Report
Source: Case Rep Anesthesiol. 2026 May 27;2026:8828001. doi: 10.1155/cria/8828001 (PMC13215977; doi:10.1155/cria/8828001)
Supplement: Supplementary file 1 — Supporting Information Supporting File 1: CARE checklist. Completed CARE checklist for the case report entitled “Noncardiogenic Pulmonary Edema After Neostigmine Administration During Emergence From General Anesthesia: A Case Report.” [file CRIA-2026-8828001-s001.docx]

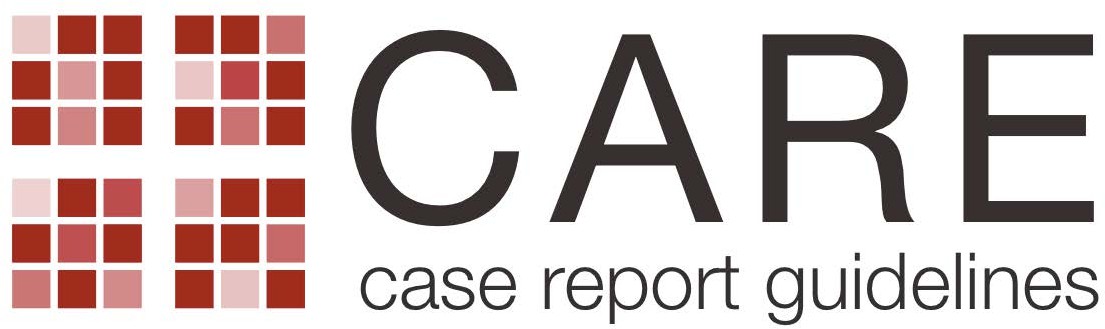
CARE Checklist of information to include when writing a case report
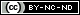


**Topic Item Checklist item description Reported on Line**

**Title 1** The diagnosis or intervention of primary focus followed by the words “case report” . . . . . . . . . . . . . . . . . . 1-2

**Key Words 2** 2 to 5 key words that identify diagnoses or interventions in this case report, including "case report" 10-11-12

**Abstract**

**(no references)**

**3a** Introduction: What is unique about this case and what does it add to the scientific literature? 29-30 / 35-36

**3b** Main symptoms and/or important clinical findings . . . . . . . . . . . . . . . . . . . . . . . . . . . . . . . . . . . . . . . . . . . . . . . . . . . 32-33

**3c** The main diagnoses, therapeutic interventions, and outcomes 33-36

**3d** Conclusion—What is the main “take-away” lesson(s) from this case? 38-39

**Introduction 4** One or two paragraphs summarizing why this case is unique (**may include** reference**s**) 54-65

**Patient Information 5a** De-identified patient specific information 75

**5b** Primary concerns and symptoms of the patient 92-93

**5c** Medical, family, and psycho-social history including relevant genetic information 77-78

**5d** Relevant past interventions with outcomes N/A

**Clinical Findings**

**Timeline**

**Diagnostic Assessment**

**Therapeutic Intervention**

**Follow-up and Outcomes**

1. Describe significant physical examination (PE) and important clinical findings 92-94/96-97
2. Historical and current information from this episode of care organized as a timeline 279

**8a** Diagnostic testing (such as PE, laboratory testing, imaging, surveys). 98-101/105-106

**8b** Diagnostic challenges (such as access to testing, financial, or cultural) N/A

**8c** Diagnosis (including other diagnoses considered) 132-138

**8d** Prognosis (such as staging in oncology) where applicable N/A

**9a** Types of therapeutic intervention (such as pharmacologic, surgical, preventive, self-care) . . . . . . . . . . . . . . . . . 95/ 103-104

**9b** Administration of therapeutic intervention (such as dosage, strength, duration) 104

**9c** Changes in therapeutic intervention (with rationale) 95/104-105

**10a** Clinician and patient-assessed outcomes (if available) 105

**10b** Important follow-up diagnostic and other test results 105-106

**10c** Intervention adherence and tolerability (How was this assessed?) N/A

**10d** Adverse and unanticipated events 102-103

**Discussion 11a** A scientific discussion of the strengths AND limitations associated with this case report 144-145/158-163/170-171

**11b** Discussion of the relevant medical literature **with references** 154-156

**11c** The scientific rationale for any conclusions (including assessment of possible causes) 165-171

**11d** The primary “take-away” lessons of this case report (without references) in a one paragraph conclusion 172-178

**Patient Perspective 12** The patient should share their perspective in one to two paragraphs on the treatment(s) they received . . . . N/A

**Informed Consent 13** Did the patient give informed consent? Please provide if requested . . . . . . . . . . . . . . . . . . . . . . . . . . . . . . . . . . . . . . **Yes X** **No
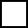
**
